# Impact of COVID-19 on people living with HIV and HIV care: A qualitative study in the Volta Region of Ghana

**DOI:** 10.1371/journal.pgph.0003017

**Published:** 2024-03-15

**Authors:** David Ayangba Asakitogum, Jerry John Nutor, Rachel G. A. Thompson, Robert K. Alhassan, Akua O. Gyamerah

**Affiliations:** 1 Department of Family Health Care Nursing, School of Nursing, University of California San Francisco, San Francisco, California, United States of America; 2 Language Center, College of Humanities, University of Ghana, Accra, Ghana; 3 Africa Interdisciplinary Research Institute, Accra, Ghana; 4 Centre for Health Policy and Implementation Research, Institute of Health Research, University of Health and Allied Sciences, Ho, Ghana; 5 Department of Community Health and Health Behavior, University of Buffalo, Buffalo, New York, United States of America; Southern Cross University, AUSTRALIA

## Abstract

The COVID-19 pandemic caused significant social changes and challenges globally, including economic slowdown and limitation of essential services. Our study explored the impact of the COVID-19 pandemic on the HIV treatment experiences and lives of people living with HIV in Ghana. Between October 2021 and January 2022, we conducted four focus group discussions with 24 people living with HIV and six in-depth interviews with healthcare providers to understand the impact of the COVID-19 epidemic on their lives and HIV treatment and care. Transcribed interviews were analyzed using thematic analysis. The COVID-19 pandemic most impacted people living with HIV economically and psychosocially. Economic challenges included loss of income/economic support, financial hardship, and material insecurities. The psychosocial impact included fear of the health impact of COVID-19 on people living with HIV and social isolation. The pandemic also impacted participants’ HIV treatment and care services including change in their site of care and non-adherence to antiretroviral therapy. Clinics in particular modified HIV care services to maintain treatment and care during the pandemic. Healthcare providers also implemented direct antiretroviral therapy service delivery to clients, which reduced patient overcrowding but increased providers’ workload and expenses. The COVID-19 pandemic caused economic hardship, social isolation, and changes in HIV treatment and care services for people living with HIV. It also imposed a work and financial burden on healthcare providers. However, service changes made by providers helped sustain HIV care and treatment for clients and should inform future pandemic responses in HIV services.

## Introduction

The COVID-19 pandemic undoubtedly brought many changes and challenges to the lives of individuals, communities, and society at large. The global impact of COVID-19 included collapsed businesses, job loss, travel restrictions, and scarcity of essential goods and services [1–3]. The pandemic similarly negatively impacted healthcare systems [4–6], including HIV care services [7–9]. For example, an online survey of 1029 healthcare providers in China on challenges of HIV services delivery during the pandemic revealed that the pandemic affected follow-up visit time, disruption in ART medication refill schedules, and patients’ non-adherence to ART [10]. These pandemic-related disruptions have significant implications for adverse health outcomes, including disease progression and opportunistic infections among people living with HIV (PLWH).

Furthermore, the COVID-19 pandemic negatively impacted HIV testing and diagnosis services [11]. A systematic review on the pandemic’s impact on HIV service and treatment outcomes [12] and a global study conducted across four continents [13] found that COVID-19 significantly decreased HIV testing, increased the percentage of positive tests [13], reduced the number of in-person medical consultations with PLWH, and showed a reduction in the number of new enrolments of patients on ART [12, 13]. These outcomes suggest that COVID-19 has disrupted the progress made in the fight against HIV in previous decades.

In another international study that surveyed 238 HIV care sites across seven geographical regions, 75% of the sites reported experiencing pandemic-related restrictions on the transportation of medical goods and service provisions, and 76% reported a negative impact of COVID-19 on clinic operations including reduced hours/day, reduced provider availability, and reduced and /or suspension of medical support from health service donors for HIV care [14]. While health care providers (HCPs) in the survey across all sites with low HIV prevalence and in high-income countries adopted telemedicine (85% to 100%) to sustain ART care, less than half of those providers at sites with a high prevalence of HIV and from low-income countries adopted mitigation measures to sustain ART care. Two percent (2%) of the low-income countries reported suspending ART clinic services [14]. Given that individuals with chronic health conditions such as HIV/AIDS were found to be at increased risk for COVID-19 infections and its related complications [6, 7], service suspension can pose a danger to the life of PLWH in low-income countries.

In sub-Saharan Africa, the COVID-19 impact was significant due to low resources and poor healthcare infrastructure [15]. For example, data from 10,855 households from Ethiopia, Malawi, Nigeria, and Uganda revealed that 77% of households lost family income and experienced increased food insecurity due to the pandemic [16]. For PLWH, the social distancing protocols further increased discrimination and stigma of PLWH [17]. PLWH further faced challenges to medication refill, adherence to clinical sessions with physicians, and ability to keep up-to-date on lab follow-ups due to the COVID-19 pandemic [18].

Data from quantitative studies also showed that COVID-19 negatively impacted the lives of PLWH and their access to the HIV care [16, 19]. However, few qualitative studies have been conducted to explore the impact of the pandemic on PLWH and HIV treatment and care. A qualitative study among young PLWH in Kenya found that PLWH experienced difficulty in accessing HIV treatment, had inadequate knowledge of COVID-19 and its relationship to HIV, and faced financial difficulties and stigma due to the COVID-19 pandemic [20].

In Ghana, a lower-middle income country with an HIV prevalence of 1.7% [21], one study on the impact of COVID-19 on ART clinic staff showed that there was erratic hospital attendance, shortages in hospital supplies, and reduced provider-PLWH engagement [22]. In another study that assessed the effects of COVID-19 on access to ART services in Ghana, ART services were not interrupted, but due to fear of contracting COVID-19 infection by seeking HIV services, PLWH in the study defaulted in adherence to treatment [23]. These two studies, however, did not evaluate the impact of COVID-19 on the lives of PLWH. There remains a gap in research on the impact of COVID-19 on HIV care and on the lives of PLWH and HCP in Ghana. To bridge this gap and recommend mitigation measures to sustain HIV care in future health pandemics, we explore the impact of COVID-19 pandemic on the lives of PLWH in Ghana and their experiences with HIV treatment and care services, as well as the impact of the pandemic on HIV services and on HIV care providers.

## Methods and materials

### Study design and setting

We used an exploratory qualitative design to assess the experiences of PLWH and HCPs during the COVID-19 pandemic. Four focus group discussions (FGDs) were conducted with 24 PLWHs and six in-depth interviews (IDI) with HCPs to gain insight into their COVID-19 experiences and the pandemic’s impact on HIV treatment and care.

The study was conducted in the Volta Region of Ghana at the Ho Teaching Hospital. The Volta Region has an HIV prevalence of 1.30% [24]. The hospital has an ART clinic that serves as the referral center for HIV treatments and care services. The ART clinic has a patient population of over 1000 PLWH. The HCPs at the ART clinic provide care to over 300 PLWH every month.

### Study population

The target population for the study was PLWH in the Volta Region or those who access ART services in the Volta Region and HCPs who provide HIV treatment and care services. To be eligible to participate, PLWH had to be 18 years or older, enrolled in ART for at least 6 months prior to the COVID-19 outbreak, access ART services in the Volta Region, and be willing to participate. Eligibility criteria for HCPs were age 18 years or older and hold a position as an allied health professional (i.e., medical doctor, nurse, or medical technician). Participants were excluded if they were seriously ill (defined as hospitalization) or had a mental impairment. More information on our study methods can be found elsewhere [25].

### Recruitment and data collection

Participants were recruited using purposive sampling with the support of an attending nurse. The attending nurse introduced the study to PLWH who attended the ART clinic to obtain their periodic ART medication refills or to consult their physicians between October 2021 and January 2022. The nurse referred potential participants who were interested to know more about the study to a research assistant. The research assistant explained the study’s purpose, benefits, risks, and patient confidentiality to the potential participants. Potential participants were informed that the study involves group discussion, and that the discussion would be audio-recorded to aid the researchers to listen to and write out exactly what was discussed. In addition, potential participants were informed that participation was voluntary and refusal to participate in the study would not affect their healthcare access in the clinic. PLWH who willingly agreed to participate in the study were given copies of the study information sheet and consent forms to sign. Participants voluntarily signed an informed consent form.

### Focus group discussions and in-depth interviews

Interview guides were piloted and revised with input from the study team prior to data collection. PLWH who signed the consent forms were informed that they would be put into groups to discuss questions regarding the changes COVID-19 has made in their lives and the way they assess ART services during the pandemic. Participants were told that there would be males-only groups, females-only groups, and mixed groups for the discussions. Participants were encouraged to indicate group preferences. The groupings were designed to have same-sex discussions freely without hiding sensitive issues themselves and to have the mixed groups discuss so that issues from same-sex discussions are probed when concealed. In all, four FGDs consisting of 5 to 7 participants in each group were formed. The groups were constituted to generate a sample that was diverse in age, gender, place of residence, and number of years diagnosed with HIV. All FGDs were conducted in a private room at the study site and facilitated by two research team members. Each focus group was asked the same questions using a focus group discussion guide designed for the study. The FGD questions for the present study were: How has COVID-19 affected your life overall? How do COVID-19 and changes related to it have affected your experiences getting HIV care? Can you tell me how COVID-19 has affected the way your HIV clinic used to do things? How have COVID-19 and changes related to it affected the way you get your HIV medication? What can be done to help you visit the clinic during health crises like COVID-19? The participants answered each question in turns. The facilitators (RKA and a research assistant) probed with follow-up questions related to participant responses whenever necessary to enrich the discussion. They also asked each member of the group to add to the discussion to ensure representation of the experiences of different participants. This approach enabled the discussion to reach data saturation, a point in which no new information was realized. The discussions were conducted in English and Ewe language and lasted for 60 to 90 minutes.

The in-depth interviews were conducted by AOG with the HCPs over Zoom using a structured interview guide. The providers were asked to share how COVID-19 affected their personal lives and ART services, as well as how the pandemic impacted PLWH clients’ treatment and care. The interviews were in English and lasted for 60 to 80 minutes.

### Data management and analysis

All interviews were audio recorded. Audios in the Ewe language were first translated into the English language by a team member who understands, writes, and speaks the language. All the interviews were then transcribed verbatim. The transcripts and audios were closely reviewed for accuracy and all inconsistencies were corrected before uploading the transcripts into Dedoose software [26]–a qualitative data management and analysis tool—for analysis.

We developed an initial codebook of a priori codes based on the interview guides for the FGDs and the in-depth interviews. The Dedoose version 9.0.17 [26] was used to manage the uploaded transcripts in organizing the codes. Using thematic analysis [26], two members of the research team (DAA and AOG) independently coded one FGD and one IDI interview transcript. The two analysts reviewed and compared their coding, discussed, and resolved differences. The codebook was revised accordingly, and all remaining transcripts were coded by DAA. AOG reviewed and cross-checked all coding with DAA and codes were grouped and/or reorganized to identify major themes and subthemes. Interview notes were also cross-referenced during the analysis to inform and confirm the accuracy of findings. The following COVID-19 data segments were further analyzed for emergent themes: 1) COVID-19 impact (parent code); 2) COVID-19 Impact on Life; 3) COVID-19 impact on clinic and services; 4) COVID-19 impact on provider HIV care; 5) COVID-19 Impact on HIV treatment/care; and 6) Recommendations: COVID-19 challenges to HIV care. The data was analyzed and displayed in tables to assess themes.

### Ethical approval

The study was conducted in accordance with the Declaration of Helsinki for research involving human subjects. The study received ethics approval from the University of Health and Allied Sciences ethical committee (# UHAS-RECA6[1]20–21) and the University of California San Francisco Institutional Review Board (# 20–32955). Permission was also granted from the management of the Ho Teaching Hospital and the HIV Clinic. Confidentiality was ensured at all stages of the process and written informed consent was obtained from the participants prior to being interviewed.

### Inclusivity in global research

Additional information regarding the ethical, cultural, and scientific considerations specific to inclusivity in global research is included in the S1 Checklist.

## Results

### Participant characteristics

There was a total of 30 PLWH and HCPs in this study (see Table 1 for participant characteristics). Among the PLWH, 14 identified as female and 10 as male. The mean age of PLWH participants was 51.7 years old (SD = 11.11). The main occupations of PLWH in this sample were trading and farming. The number of years since PLWH had been diagnosed with HIV ranged from two to 15 years. HCPs consisted of three nurses and three doctors. Their mean age was 36 years old (SD = 9.3). HCPS had a range of one and a half to seven years of work experience in their positions.

**Table 1:** Participant characteristics.

**PLWH focus group discussions (N = 24)**				
**PLWH Client**	**Gender**	**Age (Years) Mean:** 51.7 Years	**Occupation**	**Years since diagnosis**
		**SD:** 11.11		
1	Male	72	Artisan	7
2	Male	64	Security man	10
3	Male	49	Farmer	14
4	Male	64	Farmer	11
5	Male	72	Pensioner	6
6	Male	56	Security man	11
7	Male	66	Security man	7
8	Male	60	Trader	2
9	Male	47	Driver	5
10	Male	57	Farmer	7
11	Female	42	Trader	9
12	Female	46	Head dresser	13
13	Female	52	Trader	9
14	Female	38	Trader	7
15	Female	38	Pure water hacker	12
16	Female	43	Trader	12
17	Female	42	Trader	11
18	Female	47	Trader	18
19	Female	31	Seamstress	3
20	Female	39	Seamstress	6
21	Female	62	Unemployed	15
22	Female	44	Trader	14
23	Female	58	Farmer	14
24	Female	52	Trader	8
**Provider in-depth interviews (N = 6)**				
**Provider**	**Gender**	**Age (Years) Mean:** 36 Years	**Occupation**	**Years in position**
		**SD:** 9.3		
HCP 1	Female	30	Nurse	7
HCP 2	Male	26	Nurse	1.5
HCP 3	Male	39	Nurse	2
HCP 4	Female	38	Medical doctor	3
HCP 5	Female	29	Medical doctor	1.5
HCP 6	Male	54	Medical doctor	7

### Overview

Three (3) overarching themes emerged from our analysis of the impact of COVID-19 on PLWH and HIV treatment and care. These were: 1) Economic impact of COVID-19 such as loss of income/economic support, financial hardship, and material insecurity; 2) Psychosocial impact of COVID-19, such as fear of COVID-19 infection, social isolation of PLWH, and COVID-19 related stigma against HCPs; and 3) COVID-19 impact on HIV treatment and services, including changes in service provision, care adherence, and treatment dispensing/delivery, and increase in HCP workload (Table 2). The sections below elaborate on these themes in detail.

**Table 2:** Themes and subthemes on the impact of COVID-19 among PLWH and HCPs.

Themes	Subthemes	Quotes
**Economic Impact**	**Loss of business or source of financial support**	FGD 4 R4: It has affected my brothers and sisters businesses. Some are doing small businesses and when it happens like that, they used to send me some small coins, but now they always say things are not good. FGD 4 R2: For the work, we know that it has been spoilt already. Sometimes when you’re selling, you won’t get a dime. You just must pack to pack the wares. Those that are perishable would have gone bad. In that regard, Covid has spoilt things FGD4 R3: What Covid has affected me is my business. I sell pure water. At times you sell to a customer, and he’ll be like hee-hee why did you touch the water? I should pick it myself. So that is my challenge in my business.
	**Material insecurities and increased expenses**	FGD 2 R7: At that time, they said nobody is going to be allowed to cross the border to Togo, and some of us we have our farms on that side. Because of that, we could not do a proper harvest and it affected us that we don’t get food to eat. We went hungry at home and were not able to take medicines. FGD 1 R2: Some of our farms too are across the border and everything is not going well because you cannot go and bring food stuff and animals spoiled everything. So that’s how we’re living without food. So, it has affected us. FGD 2 R4: We cannot afford those masks and hand sanitizers. If you don’t have it too, they will not allow you to enter the hospital, so it was difficult to go to the clinic.
**Psychosocial Impact**	**Fear of contracting COVID-19**	FGD 4 R5: But my fear now is that anytime you feel pain in any part of your body and come here if I cough now, they say I have COVID-19. If my BP goes up just a bit now, they say I have COVID. That’s the biggest fear with people now. So even if they are sick and needed to come to see the doctor they won’t like to come. This one I won’t lie about it. You yourselves have been hearing about it. Because one of my aunties is asthmatic, when she got an attack, she was told she had Covid. So, we’re afraid. HCP 6: They don’t allow you to go home whether you are mild, moderate, or severe. So, because of that, it has put fear in the people. So, the initial approach for managing cases, especially the isolation of cases and quarantine of cases was problematic. HCP 3: During the COVID time, most of them (PLWH) they were not ready to come out especially at the initial stage most of them were not willing to come for their drugs because they were scared because they were told that those with co-morbidities or those who are having opportunistic infections they are prone to death when they contracted to COVID so those that knew they were HIV positive clients some of them were staying at home. They were not willing to come for their drugs even some of them were even calling us to, if you can bring the drugs to like their area and they will take it. Which we did.
	**Social isolation and stigma**	HCP 2: One other thing that happened was that if people get to know you are a health worker, they try to isolate themselves from you because itis believed that the infection is in the hospital and those who work at the hospital, they are the ones who carry the infection. So, it also brought a distance between erm friends and families so we couldn’t visit our families again, we couldn’t go to places we use to go at first. HCP 5: Hmm it is interesting to note that some of us were tagged as carriers of the infection and people close to us avoided our company. FGD 4 R2: it also made some of us miss our friends we used to meet and have charts with at the clinic, that’s also one change that came along with it.
**Impact on ART Services**	**Impact on HIV treatment and care**	FGD4 R7: The change that came in taking our medications is that you cannot enter the clinic without nose mask or when you enter the clinic without nose mask, they will rebuke you. If you don’t wear the nose mask well too, they will rebuke you that they only want our safety that is why they are rebuking us. So that is the only change that I see. FGD 2 R1: No, COVID-19 has not brought any changes in how we take our medication. It is same as we have been taking in the past. FGD 2 R5: Outbreak of COVID-19 has not affected the way I take medication. How I take it in the past is same way now. FGD 2 R1: Another one is that in the past, we take the medication in every three months but when we come now, you will take it for six months. So that it will keep long before you come back. HCP 4: So, the longest refill we usually give is for only three months. According to MACQ we should be able to give for six months. FGD 3 R1: Before Covid, we used to come in large numbers. We would be so many on a bench, but when Covid came, we’re told not to mass up at places, so the nurses put us in smaller groups. They call vis phone to tell us that you are for this day. So, you come for your medication and leave. Another group comes on a different date and leave. So, you’ll see that as we used to come and mass up here and stay longer before leaving had all stopped. FGD 1 R3: For me, during the Covid, formerly when we come, they made us to see the doctor, but during Covid, if there’s nothing wrong with you, you’re not allowed to see the doctor. You will not be examined. They’ll only give you your medication and then you leave.
	**Adherence to ART**	FDG 2 R1: What I’ve seen is that, even though it’s hard for us, in taking our medication, it has not affected it. If your appointment time is due, they will call you to come for the medication. FDG 2 R7: It has affected a lot of things but with regards to taking our medication, there was no problem with it. I come to take medications freely and leave. HCP Nurse 3: I can say during the COVID time, there was some, most of them were not actually coming for their drugs during the COVID time but not now. That time too a lot of them travelled to different clinic because of the lock down some of them were not also coming just because of fear. HCP 6: So, some of our clients got locked out in Togo and for Togo you must pay for anti-retroviral medications, and he was locked there for eight months.
**Recommendations**	**Provision of emergency IDs**	FGD 2 R1: So that we’re giving a card, so you can show it out to indicate that you’re going to the hospital, you’re going for medication or something like that. So, if a police officer sees you, he will allow you to go. FGD 2 R3: So as my daughter said, a form of identification should be given to us. Like TB patients also has ID. So they can allow you to go to the hospital
	**Home service—No home service**	FGD 4 R4: If they know we will not come out, then the health workers have cars, they should move from house to house and distribute the drug to us. FGD 2 R5: As there is a good relationship between the nurses and us, we can be contacting each other. How they will do to bring the medications to us, it will be fine. FGD 4 R4: … We don’t want drugs to be distributed to us in our homes for people to identify us. Finish FGD 2 R4: We don’t trust anybody in this life including us here in this room. We can’t be trusted 100%. so that is how I also see it, drugs should not be giving to us at home.
	**Provision of food and material**	HCP 4: The number one will be to give nutritional support for them (PLWH) so that it will enable them (PLWH) take the medication. FGD 1 R4: They should share nose mask, hand sanitizer and the soups that we will use to be washing our hands. They should give us those things and it will prevent us from getting the virus in addition to the one we are already suffering from. FGD 1 R6: we shouldn’t get worried but prepare ourselves and focus on living better life, eat well, and be happy. So, we need food in health crisis.

### Economic impact of COVID-19 on PLWH

#### Loss of business or source of financial support

One of the prominent themes from FGDs on the impact of the COVID-19 pandemic is that PLWH experienced job losses and/or reduced financial support from families during the pandemic. Clients expressed that family businesses were particularly affected because of COVID-19. Mitigation efforts such as lockdowns or shelter in-place that were put in place by the government also affected their movement and business transactions. This limited their sources of financial assistance to sustain their lives during the pandemic. For example, one client (FGD 4 R4) shared that the pandemic affected their siblings’ businesses, which in turn affected the client’s finances since their siblings would send them money when business was good. Another client (FGD 4 R1) described how her husband lost his hotel job during the pandemic which subsequently affected her source of economic support to maintain life.

*My husband for instance was a hotel worker and all that*. *When there was no COVID*, *work was good*. *But now*, *due to COVID*, *they closed some of those places*. *Small help you’ll ask from him now*, *because work has stopped*, *he won’t get to give you like that*.

Some PLWHs also lost their jobs during the pandemic, which caused them hardship. For example, one participant (FGD 4 R2) shared that they were unable to sell as many products as they previously did due to lower patronage at their shop. The client attributed the low demand for goods in the market to the pandemic which resulted in the loss of their perishable items “…In that regard, COVID has spoilt things and made us poorer” the client lamented.

#### Material insecurities and increased expenses

Related to the reduced sources of financial assistance to PLWH during the pandemic were material insecurities such as food instability and the increased need for pandemic-related expenses such as purchasing hand sanitizers and face masks. PLWH reported difficulty with affording food, in the acquisition of face masks and hand sanitizers, and with meeting their transportation needs during the COVID-19 pandemic. The acquisition of face masks and hand sanitizer and how to use them correctly was a major challenge because these were required for visits to the clinic.

Some participants also shared that the closure of Ghana’s borders especially with neighboring country, Togo, affected their livelihood. As one participant (FGD 1 R2) explained, due to the border closure, they could not go to their farms on the Togo side of the border, which caused the loss of farm produce and subsequently source of food and income.

### Psychological impact of COVID-19 on PLWH

#### Fear of contracting COVID-19

One of the key themes from FGDs was that many PLWHs reported being afraid of contracting COVID-19, which impacted negatively on their psychological well-being. Some of the clients stated that the fear was rooted in the fact that they were told people with comorbidities were prone to death when they contract COVID-19. They were also worried about reporting any sickness to the clinic because they believed that clinicians were misdiagnosing symptoms of other illnesses as COVID-19. Some clients even avoided sharing COVID-19-like symptoms with HCPs. As one participant (FGD 3 R2) shared, “…I told him (doctor) that everything is fine but at that time, I was not able to breathe, my nose was blocking…” In addition, one nurse (HCP 3) confirmed that some clients requested for HCPs to bring their ART refills to their areas of residence due to the fear of contracting COVID-19 in the clinical setting, “Some of them were even calling on us (HCPs) to see if you can bring the drugs to like their area and they will take it …” attributing their demand for home services to the fear of acquiring COVID-19 infection. This fear felt by PLWH during the pandemic could compromise the general health of PLWH.

#### Social isolation and stigma

HCPs reported experiences of stigma and PLWH reported experiencing social isolation during the COVID-19 pandemic. HCPs shared that people commonly believed that HCPs, especially those who were in the COVID-19 care team, were carriers of the COVID-19 infection. Due to this, doctors and nurses were isolated or were avoided, or stigmatized in their homes and communities. One nurse (HCP 2) described the isolation these changes caused, “[COVID-19] also brought a distance between us (HCPs), our friends, and our families. So we couldn’t visit our families again, we couldn’t go to places we used to go at first”. This had a significant negative psychosocial impact on HCPs who avoided their families to keep them safe from COVID-19 infection. Similarly, PLWH also felt isolated during the pandemic because they felt they would be more vulnerable to death if they got an infection. The COVID-19 social and physical distancing policies also separated PLWHs from meeting with their friends and other PLWHs at ART clinics, which deepened their psychological worries due to loneliness. As one client shared, “The coming of (COVID-19) made us sit home lonely no shaking of hands, and other people will also avoid you”.

### Impact of COVID-19 on ART services

#### Impact of HIV care/service provision

Many PLWHs shared that they did not experience significant ART service interruptions during the COVID-19 pandemic. Both clients and HCPs stated that there was a continuous supply of ART medications in their health facility. However, participants shared that the hospital did implement major changes to service provision to ensure that the risk of COVID-19 spread was mitigated at the site. For example, instead of one ART clinic day per week, the hospital added more days for clients to visit for ART pickup and HIV care, “…Another thing is that in the past, we all come and take the medication on Thursdays, but we now come on different days in the week” one client (FGD 2 R2) remarked. This then gave clients more options for days to visit the ART clinic for ART medication refills and HIV care. These changes and modifications of services also reduced overcrowding and waiting time at the ART clinic, “It has reduced the number of people that gather in the room. That is the only change that the disease (COVID-19) brought” another client (FGD 2 R4) observed. The ART medication refill time was also changed from every three months to every six months. The extension of the refill time ensured that clients had enough supply of the medication at home to prevent frequent returns to the clinic.

Despite the limited interruption to ART services, one major challenge for PLWH during the pandemic was that they had limited access to and reduced time during physical examinations by HCPs. Some PLWHs expressed that they did not receive physical examinations during the pandemic because the doctors would only conduct physical examinations for clients with major complaints. As a client (FGD 1 R3) lamented, “Formerly when we come, they made us see the doctor for examination, but during COVID if there’s nothing wrong or your problem is not serious, you are not allowed to see the doctor”.

Another notable impact the pandemic had on HIV treatment and care was related to the financial hardships PLWH clients experienced due to the pandemic. Several clients shared that the pandemic increased their expenses related to HIV care seeking because clinics required them to use masks and sanitizers to access care. One client (FGD 4 R1) expressed worry and dissatisfaction about the “no mask no entry” policy at the hospital. The client remarked, “You know they were strict on if you don’t wear a nose mask don’t enter the hospital. Hmm! It was difficult for me to buy a nose mask”. Similarly, another client (FGD 2 R4) shared that, “if you don’t wear a nose mask, you can’t enter the gate let alone enter the clinic”.

HCPs, on the other hand, experienced increased work burden due to the changes and modifications of the ART services. HCPs had to run more clinic days and deliver faster services to clients to encourage ART clinic attendance. As one nurse (HCP 3) shared about his experience,

*We have one day like every Thursday that they (PLWH) come to clinic but when COVID came*, *we had to schedule them (PLWH) like three times a week instead of once a week … we make things fast whenever they come*, *we don’t waste their time over there*.

HCPs also performed additional roles like providing health education, routine screening of all clients, and maintenance of COVID-19 protocols, particularly on infection prevention to ensure patient safety at the clinic and at home. Another nurse (HCP 1) detailed the type of COVID-19 safety information they shared with clients, “We give them health talk on COVID-19, and we make them know that COVID-19 is real. We also take them through the preventive measures [like] how they have to wear nose masks right from the house”.

#### Adherence to ART

While many clients said that the COVID-19 pandemic did not affect their ART adherence, some PLWHs shared that the pandemic caused them to default in ART and clinic attendance. One client (FDG 1 R1) explained, “I couldn’t go out to go for my drugs because of what was happening and the fear everybody was having”. In addition, PLWH who experienced food insecurity because they could not access their farm on the other side of the closed Togo-Ghana border shared that the lack of food affected their ability to take their HIV medications. As a client (FGD 2 R7), “… We went hungry at home and felt unable to take the medicines”.

Some healthcare providers shared that some clients defaulted on ART due to the lockdown/shelter-in-place policies, change of clients’ ART facility, and client avoidance of clinic due to fear of COVID-19 infection. For example, one of the HCPs (HCP 6) said, “Some of our clients got locked out in Togo (neighboring country) and for Togo, you have to pay for anti-retroviral medications, and he was locked there for eight months”. To minimize the number of defaulters during the pandemic, an officer was made responsible for tracing ART defaulters. The facility also made funds available to the officer to use to make phone calls to defaulters and some of the defaulters provided directions for the officer to deliver ART medications to their home or at preferred drop-off location. As a doctor explained (HCP 4),

*For those periods or times that we were in lockdown*, *some of them couldn’t come for their medication*, *A staff was working on them all calling people*, *getting directions to homes*, *and finding ways to send medications to them*.

### Participants recommendations

PLWHs and HCPs offered recommendations to improve HIV treatment and care provision in the context of an epidemic. PLWHs particularly recommended that health clinics provide their clients with emergency identification cards during a health crisis. They expressed doing so would enable them to have special access to visit the clinic during a lockdown and continue accessing their services. One client, (FGD 2 R1) explained, “With an ID, if a police officer sees you, he will allow you to go [and seek medical attention]”. Another notable recommendation that PLWHs were divided on was the dispensing of ART medication at home or in a community setting to improve access during a health crisis like the COVID-19 epidemic. While some clients wanted ART home services because of the good relationship they have with their HCPs, others opposed ART home services because they feared receiving services at home would compromise confidentiality and disclose their HIV status to their families and community members.

In addition, given the economic impact of COVID-19 on PLWHs, both PLWHs and HCPs recommended that clinics or government agencies should provide PLWHs with free food and other essential materials like PPEs to PLWHs to ensure they are well protected and nourished. Thus, they will be able to take their medications without side-effects and access health services under pandemic conditions. As HCP 4 remarked, the most important thing to do for PLWHs during a health crisis is “the provision of nutritional support”.

## Discussion

The current study explored the impact of the COVID-19 pandemic on the lives of PLWH and their experiences with HIV treatment and care services, as well as the COVID-19 related experiences of HIV care providers in Ghana. Our findings indicate that the pandemic had a major economic impact on PLWH such as the loss of their businesses and other sources of economic support and sustenance, as well as increased expenses due to COVID-19 related needs of PLWH clients. We also found that the pandemic had a negative impact on the psychosocial wellbeing of PLWH and HCPs, such as fear and worry about contracting COVID-19 infection, social isolation due to the pandemic, and COVID-19 related stigma experienced by HCPs due to their role as frontline workers. Our findings further revealed that while there was no HIV service and care disruption, PLWH found that the extent of their care, such as duration of physical examination by HCPs, was compromised during the pandemic because HCPs were worried about contracting COVID-19 during these examinations, while some reported experiencing an increased workload. Additionally, PLWH reported non-adherence to ART due to the pandemic. These findings highlight significant social and health care challenges experienced due to the COVID-19 epidemic that should inform pandemic preparedness planning within HIV service provision, particularly the development of mitigation strategies and the enhancement of COVID-19 protocols in HIV care in preparation for future pandemics.

Consistent with previous reviews on the impact of COVID-19 on the socioeconomic lives of people generally [1–3] and among PLWH [7–9], in our study, PLWH lost their business and/or sources of financial support from family and friends. This exacerbated economic hardship in the lives of PLWH who already face economic hardship due to the impact of HIV/AIDS [27]. We also found that PLWH experienced food insecurity and even starvation because of economic hardship and inability to access food from their farms due to the closure of the Ghana-Togo border. The issue of food insecurity is particularly important to further explore because, as a study in Uganda found, increased depression among PLWHs during the COVID-19 pandemic was associated with food insecurity [28]. Moreover, food insecurity has been found to negatively impact HIV treatment and adherence [29–31]. Unfortunately, although the Government of Ghana had a free food distribution program during the country’s COVID-19 lockdown, the program did not target vulnerable populations such as PLWH [32, 33]. In addition, our finding that the healthcare facility (ART Clinic) incurred increased expenditure for HIV services and care during the pandemic means that during the epidemic, there were few or no financial resources allocated to critical healthcare services such as ART, TB, and Malaria in the country. We recommend a targeted food and resource distribution program to include persons living with chronic health conditions (e.g., PLWH), and immediate allocation of funds to critical healthcare programs (i.e., ART, TB, Malaria, Immunizations) in future health crises.

Another key finding is that similar to other studies, PLWH feared contracting COVID-19 due to their increased comorbidity risk for COVID-19 infection [7]. We also found that HCPs were afraid of transmitting COVID-19 to their families, which has also been reported elsewhere [34, 35]. These fears and concerns caused HCPs and PLWH to feel socially isolated, consistent with other studies’ findings [8, 36]. One notable source of isolation for PLWH was the physical distancing policy and the increased ART clinic days from one day to three days per week which prevented PLWH from meeting their colleagues at the clinic attendance.

In addition, our finding that some PLWH experienced isolation during the pandemic is partially consistent with other studies. In our study, the participants felt isolated because of the physical distancing policy and the increased ART clinic days from one day to three days per week which prevented PLWH from meeting their colleagues at the clinic attendance. In addition, and consistent with a previous review [35] and in one study in Ghana [22], HCPs were avoided, stigmatized, and discriminated against during the pandemic. The main reason for the discrimination of HCPs was the belief that HCPs were carriers of COVID-19 infection and thus, the community and family members avoided contact with them. The creation of COVID-19 camps other than the traditional hospital settings further deepened this myth. Healthcare policymakers should anticipate such myths and stigmas and develop messages to dispel these myths in future health crises. There should also be a post-COVID-19 psychological assessment of HCPs who worked directly with COVID-19 cases or were actively involved in COVID-19 contact tracing to identify any post-traumatic disorders among the CHPs.

Inconsistent with findings from other studies [10, 37] that reported serious HIV treatment and care disruptions due to COVID-19, in our study, both PLWH and HCP experienced no significant HIV service and treatment supply disruptions. This is likely because in response to COVID-19, the Ghana AIDS Commission modified HIV services by increasing ART medication refill schedules from 3-month supply to 6 months. This ensured the uninterrupted ART medication supplies to PLWH [23, 38]. In addition, and consistent with previous studies in Africa [18] and one study in Ghana [23], in the current study, few PLWHs reported non-adherence to ART. The main reasons for ART default as reported in our study were PLWH’s inability to acquire face masks or transportation fees, and their fear of contracting COVID-19 during a clinic visit.

Another key impact of COVID-19 on HIV care is that some PLWH observed shorter physical examinations from HCPs during the epidemic, a finding that is supported by previous studies [13, 14, 39]. However, the reasons why physical examinations were not conducted for PLWH differ. In our study, HCPs claimed clients’ complaints were not serious enough to warrant physical examination, while in other studies [14, 39], there was reduced consultation time and schedules that prevented physical examination of PLWH during the epidemic or physicians were simply inaccessible. The consequences of ignoring PLWH medical complaints by not conducting physical examination could be dire, and clinicians need to be attentive to medical complaints of PLWH during future health crises. Clinics can enable this by ensuring that hospitals are adequately staffed. As HCPs in our study reported, there was an increased workload during the epidemic, including COVID-19 education, organizing and participating in weekly meetings and trainings, and increased working hours without compensation. This finding is similar to findings from a qualitative study from Kenya [37] but inconsistent with another study where HCPs’ work schedules and time spent with PLWH were drastically reduced [14].

### Study recommendations

Our findings offer several recommendations that can help Ghana and other countries incorporate HIV prevention and care services sustainability plans in future health crises and emergency preparedness. Similar to the COVID-19 mitigation program in Zambia on HIV treatment [38], we recommend six months of ART supplies to PLWH, tracing ART defaulters, and providing them with ART supplies in future health crises. Measures like these have been documented to significantly increase PLWH early return to ART clinics and reduce interruptions in HIV care to PLWH in Zambia [38], during the COVID-19 pandemic.

For the ART clinic attendance schedule, we observed that the increase in clinic days was positively received by PLWH clients and should be maintained post-pandemic because it reduced the number of people in the clinic at a given time, and thus, clients’ waiting time. Regarding the dispensing of ART medication to PLWH, we found that clients have different preferences for accessing treatment due to fear of HIV stigma. We thus recommend offering clients three dispensing approaches: dispensing at the ART clinic, dispensing at home or at the client’s preferred meeting point, and dispensing at a Health Centre near the client. Depending on the choice of the clients, the implementation of this dispensing arrangement can ensure ART service continuity in future health crises.

We also recommend that the government and civil society organizations provide socio-economic empowerment programs to support PLWH. Programs such as free or subsidized food distribution to PLWH, disbursement of stipends to PLWH for food and transportation, and provision of essential materials to help support PLWH clients who have limited sources of income to sustain their livelihood—an important factor for optimal HIV treatment outcomes.

Finally, in terms of health system recommendations, the government through the Ministry of Health should expand their healthcare work staff, especially in preparation for future pandemics when the need increases to support the increased workload. We also recommend a post-COVID-19 psychological assessment of HCPs who worked directly with COVID-19 cases to identify and manage any post-traumatic disorders.

### Study limitations

First, our study utilized a qualitative research approach in which participants were purposively sampled, thus findings cannot be generalized to all PLWH and HCP in Ghana. Second, due to the onset of the COVID-19 pandemic, we were unable to conduct member checking to validate study findings. Third, our sample was an average older PLWH who are engaged in care, which may reflect clients who have been more adherent to treatment and care. Thus, experiences shared may not be generalizable to younger PLWHs. Despite these limitations, our study makes an important contribution to the literature on understanding the impact of COVID-19 on PLWH and HCPs.

## Conclusion

Our study found that both PLWHs and HCPs experienced significant challenges such as economic hardship, psychosocial distress, and changes to HIV services due to the COVID-19 epidemic in Ghana. HCPs experienced increased workload and financial burdens due to service changes implemented to sustain HIV care during the pandemic, while PLWH experienced food insecurities due to the socioeconomic impact of the epidemic. While the COVID-19 pandemic did not interrupt HIV services and care for PLWH in our study, they observed reduced care time with HCPs. These findings contribute important information that should inform the strengthening of Ghana’s pandemic preparedness protocol and practices for future health crises.

## Supporting information

S1 Checklist Inclusivity in global research questionnaire. (DOCX)

S2 Checklist COREQ (COnsolidated criteria for REporting Qualitative research) checklist. (PDF)
